# Imaging genetics approach to Parkinson’s disease and its correlation with clinical score

**DOI:** 10.1038/srep46700

**Published:** 2017-04-21

**Authors:** Mansu Kim, Jonghoon Kim, Seung-Hak Lee, Hyunjin Park

**Affiliations:** 1 Department of Electronic, Electrical and Computer Engineering, Sungkyunkwan University, Korea; 2 Center for Neuroscience Imaging Research, Institute for Basic Science, Korea; 3 School of Electronic and Electrical Engineering, Sungkyunkwan University, Korea

## Abstract

Parkinson’s disease (PD) is a progressive neurodegenerative disorder associated with both underlying genetic factors and neuroimaging findings. Existing neuroimaging studies related to the genome in PD have mostly focused on certain candidate genes. The aim of our study was to construct a linear regression model using both genetic and neuroimaging features to better predict clinical scores compared to conventional approaches. We obtained neuroimaging and DNA genotyping data from a research database. Connectivity analysis was applied to identify neuroimaging features that could differentiate between healthy control (HC) and PD groups. A joint analysis of genetic and imaging information known as imaging genetics was applied to investigate genetic variants. We then compared the utility of combining different genetic variants and neuroimaging features for predicting the Movement Disorder Society-sponsored unified Parkinson’s disease rating scale (MDS-UPDRS) in a regression framework. The associative cortex, motor cortex, thalamus, and pallidum showed significantly different connectivity between the HC and PD groups. Imaging genetics analysis identified PARK2, PARK7, HtrA2, GIGYRF2, and SNCA as genetic variants that are significantly associated with imaging phenotypes. A linear regression model combining genetic and neuroimaging features predicted the MDS-UPDRS with lower error and higher correlation with the actual MDS-UPDRS compared to other models using only genetic or neuroimaging information alone.

Parkinson’s disease (PD) is a progressive neurodegenerative disorder characterized by bradykinesia, resting tremors, rigidity, and difficulty with voluntary movement^1^. Typically, PD is diagnosed using criteria such as the United Kingdom’s Parkinson’s Disease Society Brain Bank^2^. The pathological hallmark of PD is the loss of dopaminergic neurons in the substantia nigra (SN) and those that project into the striatum. This neuronal loss leads to dopamine imbalance, which may result in inhibition of basal ganglia output and dysfunction within the cortico-basal ganglia-thalamocortical (CBGT) circuit^3^,^4^. Environmental toxins and genetic factors have been linked to the development of PD^5^. More than 16 PD-related genetic mutations have been recognized^6^.

Various neuroimaging approaches have been used to investigate how PD affects the brain^4^,^7^,^8^,^9^,^10^. These imaging approaches include positron emission tomography (PET), single-photon emission computed tomography (SPECT), and magnetic resonance imaging (MRI). Functional dopaminergic imaging such as dopamine transporter SPECT has been widely used to assess nigrostriatal degeneration^11^. White matter integrity could be assessed using diffusion MRI and tractography, which allows investigation of neuronal degeneration^12^. These various neuroimaging approaches allow us to better understand the neurobiological mechanism of PD^13^.

Existing neuroimaging studies related to the genome of PD have focused on certain candidate genes^14^,^15^. Recently, genome-wide association study (GWAS) has been used to identify genetic polymorphisms that influence disease phenotypes^16^. Common genetic variants that are associated with many diseases such as Alzheimer’s disease, PD, and diabetes have been successfully identified using GWAS^16^,^17^. Genes associated with PD have been identified using GWAS^6^,^18^. GWAS is frequently limited by the need for a large number of data samples to achieve statistical significance. Sample size requirements can be reduced using quantitative trait analysis, a form of genetic analysis that uses intermediate phenotypes of the disorder being studied^19^. Imaging genetics is a type of quantitative trait analysis that uses imaging measures (e.g., functional connectivity and brain activation) that are heritable and correlated with the disease in question.

Imaging genetics is an emerging tool that is capable of identifying the genetic risk of a disease based on both clinical factors and imaging techniques^20^. Imaging techniques provide complementary high dimensional information regarding the disease, and thus, GWAS could be improved by such additional information. Existing imaging genetic studies have been applied to Alzheimer’s disease and schizophrenia^17^,^19^,^21^,^22^,^23^. However, the application of imaging genetics for PD has been scarce. Prior imaging genetics studies have focused on specific gene candidates and have not used imaging measures for quantitative trait analysis^14^,^15^. One study investigating the cause of familial PD using functional PET imaging and genetic data reported that a mutation in the LRRK2 gene led to familial PD^14^. Another study investigated the early progression of PD in patients with mutant Parkin and PINK genes using functional MRI (fMRI)^15^. They showed that Parkin mutation carriers had abnormalities in the supplementary motor area and premotor cortex during a simple sequence task and that PINK mutation carriers were affected in the same regions during a finger sequence task.

In our study, we applied imaging genetics combining genetic polymorphisms and brain connectivity measurements as intermediate phenotypes to identify clinically relevant genetic variants in PD. Another aim of our study was to determine if the use of genetic variants along with their associated neuroimaging features could better predict clinical scores of PD progression compared to using conventional approaches with GWAS or neuroimaging alone. We obtained diffusion MRI and DNA genotyping data from the Parkinson’s disease Progression Marker Initiative (PPMI) database^24^. Connectivity analysis was used to identify neuroimaging features within the CBCT circuit that could differentiate between healthy control (HC) and PD groups. These neuroimaging features and associated clinical scores were used as intermediate phenotypes in imaging genetics analysis to identify clinically relevant genetic variants. We then analyzed the utility of combining different genetic variants and neuroimaging features for predicting the Movement Disorder Society-sponsored unified Parkinson’s disease rating scale (MDS-UPDRS), a well-established measure reflecting motor symptoms and clinimetric properties of PD, using a regression framework^25^.

## Results

### Subject selection

First, quality control of genotype data was performed based on the Enhancing Neuro Imaging Genetics through Meta Analysis (ENIGMA) protocol. In total, single-nucleotide polymorphisms (SNPs) with a minor allele frequency <1% (n = 53,482), Hardy-Weinberg equilibrium <10^−6^ (n = 683), and a genotype missing rate >5% (n = 14,378) were excluded from the study. Following quality control, 136,350 SNPs remained for further analysis. Second, the population stratification of the genetic data was adjusted using multidimensional scaling. After population stratification, only unrelated Caucasian subjects (n = 418) remained (Supplementary Figure S1). Of these unrelated Caucasian subjects, 146 (HC = 40, PD = 106) had both neuroimaging and genetic data available in the database. Forty of these PD cases were then randomly chosen to achieve equal numbers of samples in each group.

### Connectivity differences

Differences between the HC and PD groups were quantified in terms of degree centrality (DC) values using t-tests with Bonferroni correction. Four regions, namely the associative cortex, motor cortex, thalamus, and pallidum, had significantly different DC values between the HC and PD groups (Table 1). The associative cortex, motor cortex, and thalamus showed increased DC values in PD patients compared with HC subjects, while the pallidum showed reduced DC values in PD patients compared with HC subjects.

### Genetic variants in imaging genetics analysis

The results of imaging genetics analysis using the DC values of the associative cortex, motor cortex, thalamus, and pallidum and MDS-UPDRS are shown in Table 2. Three SNPs were associated with the DC values of these four regions, and four SNPs were associated with the MDS-UPDRS. PARK2 (which contained three SNPs) was associated with the DC value of the associative cortex and MDS-UPDRS. PARK7 was associated with the DC value of the motor cortex, and SNCA was associated with the DC value of the thalamus. HtrA2 and GIGYF2 were associated with the MDS-UPDRS.

### Linear model analysis

We compared three multiple linear regression models to determine which model best explained the MDS-UPDRS. Stepwise regression was applied to construct three linear regression models, and the process led to different regression models depending on the initial model. *Model C* (the one with both genetic variants and neuroimaging features) (3) showed the best performance (adjusted R-squared (adj-R^2^) = 0.714, p < 0.001) compared to *model A* (the one with neuroimaging features) (1) and *model B* (the one with genetic variants) (2), which yielded adj-R^2^ values of 0.508 (p < 0.001) and 0.442 (p < 0.001), respectively (Tables 3,4 and 5). Details regarding the three equations are found in the Methods section. Specifically, the DC value of the pallidum singly explained the greatest (9.70%) variance (F = 21.52, p < 0.001) in *model C* (3). *Model C* (3) explained 72.51% of the variance in the MDS-UPDRS, while *model A* (1) and *model B* (2) explained 53.25% and 42.74% of the variance in the MDS-UPDRS (Tables 3,4 and 5). Thus, combining genetic variants with neuroimaging features resulted in an improved adj-R^2^ value, indicating that they provide complementary information. Each model included a distinct set of independent variables (IVs), and thus, the number of included IVs varied from model to model. A given variable might appear in two different models, but its contribution in terms of percentage of explained variance could be different as there was a distinct set of IVs in each model. For example, the DC of the thalamus explained 10.75% of the variance in conjunction with 5 other IVs in *model A*, while the DC of the same region explained 6.87% in conjunction with 9 other IVs in *model C*.

### Prediction of MDS-UPDRS

**Figure 1:**
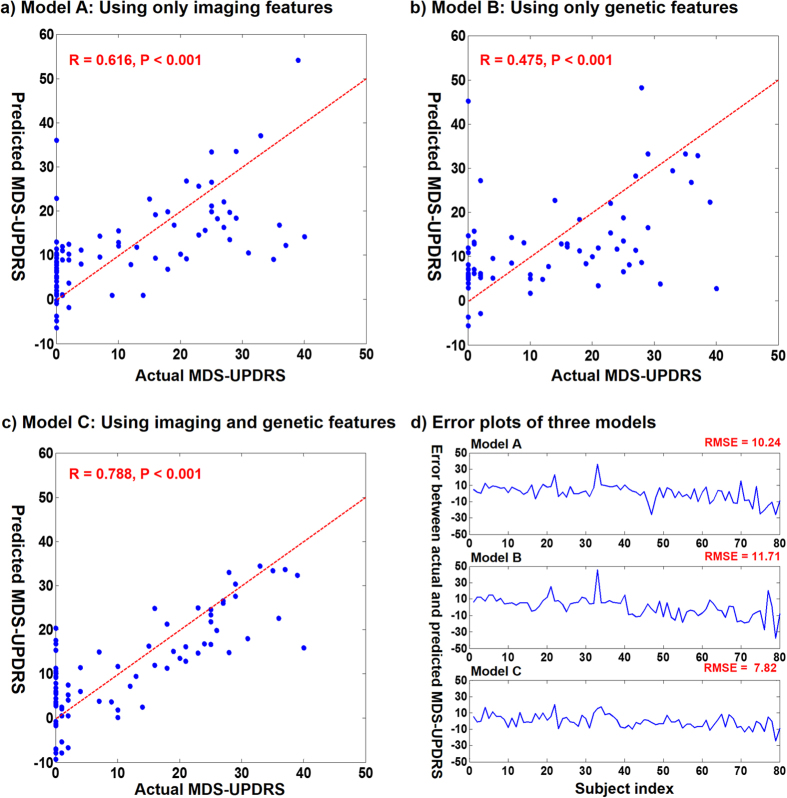
Plots of actual and predicted MDS-UPDRS for three prediction models. Sub-figures (**a,b** and **c**) show actual and predicted MDS-UPDRS from *Models A* (1)*, B* (2), and *C* (3), respectively. The dashed line indicates the identity line (i.e., actual score = predicted score). (**d**) shows error plots of the three models.

The model combining genetic and neuroimaging features, *model C* (3), predicted the MDS-UPDRS with root mean squared error (RMSE) of 7.82 and showed a high correlation with the actual MDS-UPDRS (r = 0.788, p < 0.001) compared with the models using genetic and neuroimaging features alone (*model A* (1) and *model B* (2)). The first model with only neuroimaging features, *model A* (1), showed a high RMSE of 10.24 and a correlation of 0.616 (p < 0.001). The second model with only genetic variants, *model B* (2), yielded a RMSE of 11.71 and a correlation of 0.475 (p < 0.001). Plots of the actual and predicted MDS-UPDRS scores for these three models are given in Fig. 1.

## Discussion

Our main finding showed that the linear prediction model combining neuroimaging and genetics could explain 72.51% of the variance in the MDS-UPDRS, and the model resulted in a high correlation (r = 0.788) between the predicted and actual MDS-UPDRS. Our connectivity analysis revealed that the associative cortex, motor cortex, thalamus, and pallidum had significantly different DC values between the HC and PD groups. Imaging genetics analysis identified PARK2, PARK7, HtrA2, GIGYRF2, and SNCA as genetic variants that are significantly associated with intermediate phenotypes. These findings illustrate that the identified PD-related genes might yield structural connectivity changes that could be recognized using diffusion MRI within the CBGT circuit. The linear regression model combining genetic and neuroimaging features predicted the MDS-UPDRS with a lower RMSE and higher correlation with the actual MDS-UPDRS compared to the other models using only genetic or neuroimaging information alone.

Many recent neuroimaging studies have investigated PD using brain connectivity^4^,^8^,^10^,^26^,^27^. Connectivity analysis assumes that the brain is a complex interconnected network, and it is suitable for exploring large-scale circuits that include the cortical-thalamic and cerebro-cerebellar circuits^28^. Connectivity could be assessed using centrality measures including betweenness, eigenvector, degree, and page-rank centrality to assess network properties in the brain^29^. Each centrality characterizes the importance of a given node in terms of global or local contribution to the network. There is no single centrality measure that works well in all network assessments, and the optimal centrality measure is different depending on the imaging modality and the disease of concern^30^. DC has a straightforward neurobiological interpretation in structural connectivity networks, and thus, we utilized DC in this study. Our connectivity analysis revealed that the associative cortex, motor cortex, thalamus, and pallidum had significantly different DC values between the HC and PD groups. Some previous studies also showed that these regions were important in PD patients based on fMRI, diffusion MRI, and SPECT^8^,^27^,^31^,^32^,^33^.

Conventional genetic analyses have identified genetic variants linked with PD^5^,^18^. Imaging genetics combines imaging information with genetic information, allowing for identification of additional genetic variants^22^. Thus, the analysis of PD with imaging genetics might identify additional information to better investigate the neurobiological mechanisms of PD. In this study, an effect of PARK2 (Parkin) was observed on the DC value of the associative cortex and clinical scores. PARK2 plays an important role in presynaptic and postsynaptic neuronal processes^34^. Mutations in the Parkin protein are responsible for disrupting E3 ubiquitin ligase activity in PD^35^. The associative cortex plays a secondary role in processing motor information in the classic model of the CBGT motor circuit^31^. Previous work showed that a mutation of PARK2 was associated with activity in the left lateral orbitofrontal cortex, which is part of the associative cortex^36^. Another study reported that the DC value of the dorsolateral prefrontal sub-region of the associative cortex was increased in PD patients compared to HC using resting state-fMRI^8^. These findings suggest that PARK2 may be linked with connectivity in PD patients and in turn may affect the MDS-UPDRS. An effect of PARK7 (DJ-1) was observed on DC values in the motor cortex. PARK7 is involved in transcriptional regulation and response to oxidative stress^37^. The motor cortex is a key region involved in motor processing, serving as a major input into the basal ganglia^1^,^13^. Our findings suggest that PARK7 may affect neurons in the motor cortex and in turn may affect PD symptoms. Previous reports showed that characteristic PD findings, such as dopaminergic neuronal death in the SN and motor abnormalities, are affected by mutations in PARK7^37^. One resting state-fMRI study showed that connectivity between the putamen and motor cortex increased in PD patients^27^. Another study reported that the DC value of the parietal cortex, a sub-region of the motor cortex, was increased in PD patients compared with HC subjects^8^. Moreover, one diffusion MRI study reported that track density in the primary somatosensory cortex was increased in PD patients compared with HC subjects^32^. Thus, the physiologic association of PARK7 with neurons in the motor cortex suggested by our results could be feasible. The interaction term between rs9346876 (PARK2) and rs363611 (HtrA2) led to 15.28% and 9.11% of explained variance for *models B* and *C*, respectively, in the linear regression analysis. This finding is consistent with previous studies reporting that HtrA2 specifically binds to and directly cleaves the E3 ubiquitin ligase PARK2^38^,^39^.

We performed additional analysis with 14 right-sided and 14 left-sided onset cases in the PD group using contralateral ROIs, and detailed results are shown in the supplemental material. We observed better results for the reduced 28 patient group compared to using the previous 40 patients in terms of adj-R^2^ values. *Model C*’s adj-R^2^ improved from 0.714 to 0.768, *model A*’s adj-R^2^ improved from 0.508 to 0.650, and *model B*’s adj-R^2^ improved from 0.422 to 0.485. Similar to the main results with 40 patients, *model C* (adj-R^2^ = 0.768) outperformed models A (adj-R^2^ = 0.650) and B (adj-R^2^ = 0.485) implying that combining genetic variants with neuroimaging features indeed was the best model. Similar to the main results, for the reduced 28 patients, *model C* showed the best prediction performance with low RMSE (RMSE = 3.35) and a high correlation with the actual MDS-UPDRS (r = 0.847, p < 0.001).

Our study was limited by the small number of available samples. This issue was partly mitigated by analyzing SNPs and genes previously linked with PD^6^. Our work focused on known genetic variants, thereby establishing a foundation for future research. The neuroimaging data in our study came from nine different clinical sites, but all nine sites adopted the same image acquisition protocol using the same MRI scanner model (Siemens 3T scanner) to reduce confounding effects. In addition, we added multi-center information as a nuisance co-variate to account for multi-center confounding effects. We restricted the connectivity analysis to eight known regions within the CBGT circuit, which did not cover the entire brain. We used a single imaging modality (i.e., diffusion MRI) to compute intermediate phenotypes of PD. A multi-modal imaging study, potentially including PET and SPECT, could provide complementary information and thus has the potential to provide further insight into the pathophysiology of PD. Finally, we retrieved data from a research database that lacked follow-up longitudinal data. Future work should consider longitudinal data to test the validity of our models.

## Methods

### Subjects and imaging acquisition

This study was a retrospective analysis of anonymized data, and institutional review board (IRB) approval was obtained at Sungkyunwkan University. Our study was performed in full accordance with the local IRB guidelines. Informed consent was obtained from all subjects. The baseline neuroimaging (T1-, T2-, and diffusion MR) and DNA genotyping data were obtained from the PPMI research database^24^. In detail, diffusion MRI scans were acquired with the following acquisition parameters: TR = 900 ms, TE = 88 ms, b = 1000 s/mm^2^, 64 diffusion gradient directions with one b0 image, image matrix = 116 × 116 × 72, and voxel resolution = 1.98 × 1.98 × 2 mm^3^. T1-weighted MRI scans were acquired using the following parameters: TR = 2300 ms, TE = 2.98 ms, TI = 900 ms, image matrix = 240 × 256 × 176, and voxel resolution = 1 × 1 × 1 mm^3^. T2-weighted MRI scans were acquired using the following parameters: TR = 3000 ms, TE = 101 ms, image matrix = 228 × 256 × 48, and voxel resolution = 0.9375 × 0.9375 × 3 mm^3^. The PPMI database included 423 PD and 196 HC cases as of July 2015. PD cases were classified using the criteria established by the PPMI consortium^24^. Subjects who underwent both MRI imaging and genetic testing were considered for this study. We used off-medication subjects so that the effects of levodopa therapy were excluded. Additional patient data including MDS-UPDRS, Hohn and Yahn, Geriatric Depression Scale (GDS), Montreal Cognitive Assessment (MoCA), handedness and family history were collected for each case at the baseline visit (Supplementary Table S1). Population stratification of genetic data was adjusted using multidimensional scaling. We used multidimensional scaling analysis based on the ENIGMA protocol to reduce the population stratification and to ensure genetic homogeneity for all subjects^40^. All quality controlled SNPs were compared to the HapMap3 reference population using Plink v1.09 software^41^. Identity by state distance was calculated for all autosomal SNPs so that only unrelated Caucasian subjects remained. Of these unrelated Caucasian subjects, we chose 40 HC and 40 PD cases as shown in the Results section. The age, sex ratio, handedness, and cognitive assessments of the HC and PD groups were matched (Supplementary Table S1). Among 40 PD patients, 14 were right-sided, 14 were left-sided, and 12 were bilateral onset cases.

### Tractography

**Figure 2:**
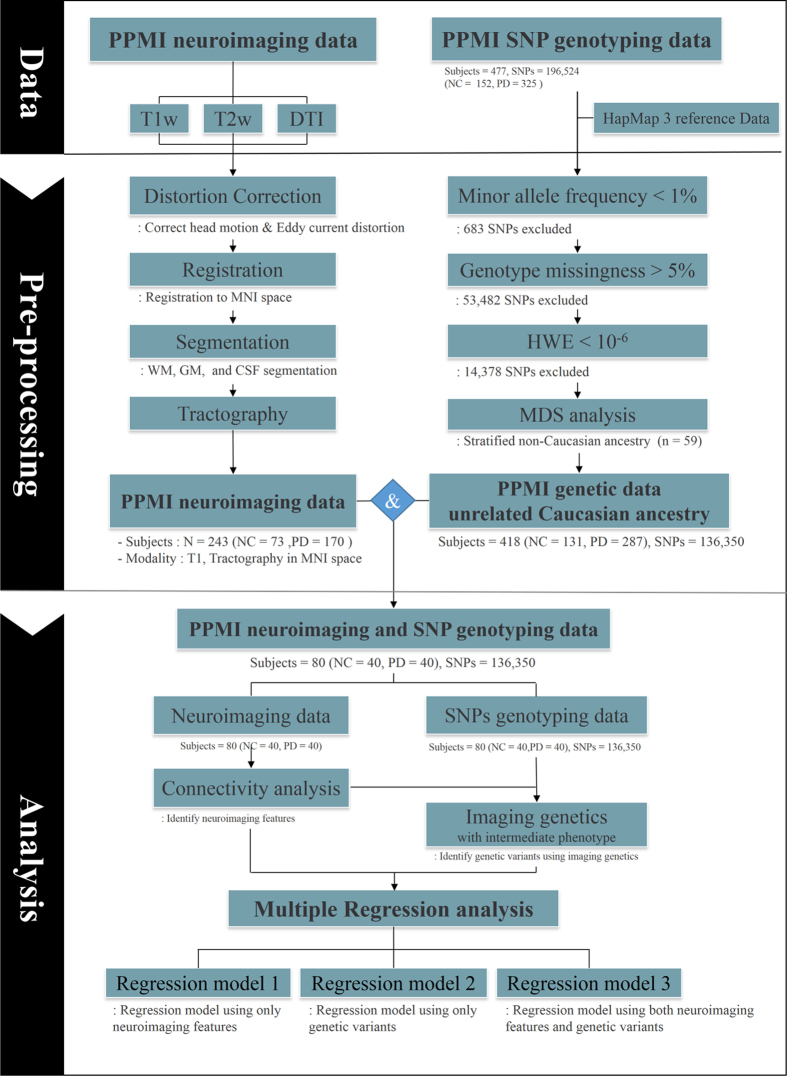
Overview of neuroimaging and image genetics processing steps.

Diffusion MRI data were processed with tractography, an algorithm that can extract *in vivo* neuronal fiber information, as described in Fig. 2. In brief, distortions due to eddy currents and head motion were first corrected using FSL. Second, T1-, T2-, and diffusion-weighted MRI scans were aligned to the Montreal Neurological Institute space with non-linear registration using FSL^42^. Then, white matter, gray matter, and cerebrospinal fluid were segmented from the registered T1-weighted image using Freesurfer^43^. The segmented white matter was used to guide the tractography algorithm. Fiber information was computed using the deterministic tractography algorithm implemented in the Diffusion Toolkit^44^. For connectivity analysis (performed with MATLAB), structural connectivity matrices were constructed using a deterministic tractography algorithm.

### Region of interest specification

**Figure 3:**
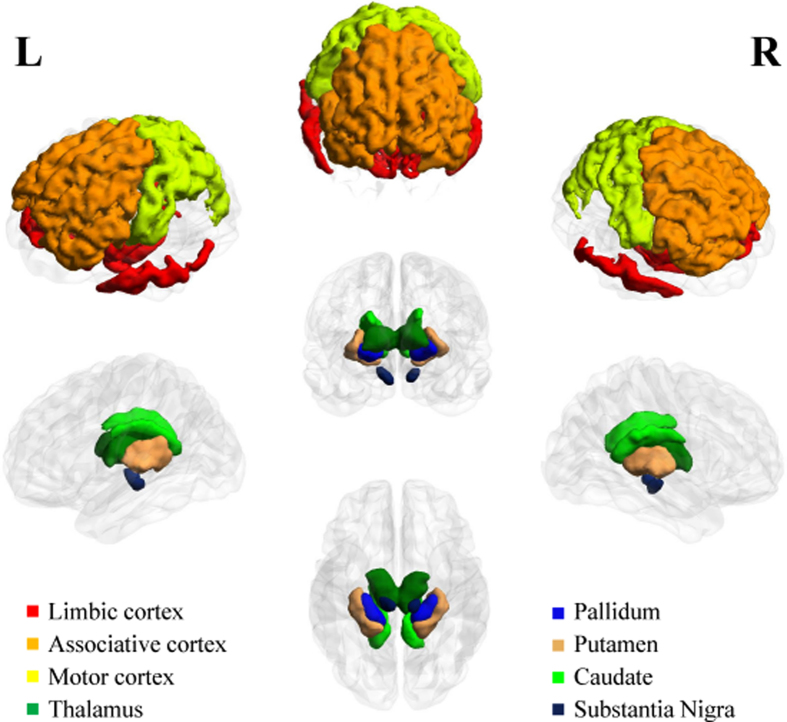
ROI specifications. Eight ROIs associated with PD in the CBGT circuit were defined based on MR images. The ROIs include the associative cortex, limbic cortex, sensorimotor cortex, caudate, putamen, pallidum, thalamus, and substantia nigra.

Connectivity analysis requires nodes and edges in a graph structure. Eight ROIs associated with PD were adopted from the existing literature and used as connectivity nodes^4^. The eight ROIs were the caudate, putamen, pallidum, thalamus, sensorimotor cortex, associative cortex, limbic cortex (specified by the Desikan-Killiany atlas) and SN (specified by transferring ROI information from a pre-defined atlas via image co-registration) (Fig. 3)^45^,^46^. These regions are members of the CBGT circuit and are known to be associated with motor, executive, and movement functions^1^,^4^,^47^,^48^.

### Structural connectivity analysis

Structural connectivity was computed using eight regions as nodes and the fiber density between two regions as edges. The fiber density between two regions was defined as follows: 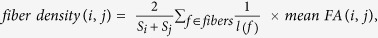 where f is the fiber connection between regions i and j, *l*(f) is the length of the fiber connection, S_*i*_ is the surface area of region i, and mean FA(i, j) is the mean fractional anisotropy value along all fiber connections between regions i and j. In other words, fiber density was calculated as the connection efficiency between two regions, which was further defined as the number of fibers normalized by length and surface area^49^,^50^,^51^,^52^,^53^. The edge values were entered as elements of the matrix, referred to as the connectivity matrix. A simple network model that considered undirected and weighted edges was used to construct the brain network. We adopted DC, which is a simple and sensitive measure of structural connectivity, to measure the properties of the brain networks^30^. DC was computed as the sum of all edge weights connected in a given node^54^. DC represents the importance of a given node, where a high DC value signifies high information flow in the node. The microstructure of white matter is under strong genetic control, and thus, the white matter structural connectivity might be affected by genes^55^.

### Group-wise differences in neuroimaging

Group-wise differences in neuroimaging were assessed with t-tests and Bonferroni correction between the HC and PD groups. For each group, the DC values of the eight nodes were stacked into three-dimensional matrices. Each group had a single three-dimensional matrix. Each element in the stacked connectivity matrix contained 40 observations. We performed t-tests to compare between the HC and PD groups to assess group-wise differences. Multiple comparison issues were corrected using the Bonferroni correction (p < 0.05, corrected)^56^,^57^. DC values of regions with significant differences were used as intermediate phenotypes for imaging genetics.

### Genetic quality control

We quality controlled DNA genotyping data based on the ENIGMA protocol^40^. Quality control of genotype data was performed based on minor allele frequency, genotype missing rate, Hardy-Weinberg equilibrium, and genotyping rate. SNPs that did not satisfy the criteria were excluded, and subjects with a low genotyping rate were also excluded. In total, SNPs with a minor allele frequency <1%, Hardy-Weinberg equilibrium <10^−6^, and a genotype missing rate >5% were excluded from the study. Following quality control, the remaining SNPs were used for further analysis.

### Imaging genetics

Imaging genetics analysis was conducted using intermediate phenotypes to identify genetic variants associated with PD. In the first analysis, neuroimaging features (i.e., DC values of regions with significant differences between HC and PD) were used as intermediate phenotypes. In the second analysis, the clinical score (i.e., MDS-UPDRS) was used as an intermediate phenotype. The genetic variance was described with an additive model^58^. Imaging genetics was performed using Plink software^41^. A linear regression analysis was performed at each SNP using the number of minor alleles as the independent variable and various types of intermediate phenotype information as the dependent variable. Age and sex were included as covariates in the linear model. Significant genetic variants were determined using a permutation test by performing random group assignments 10,000 times^23^,^59^. We focused on SNPs that were previously linked with PD.

### Construction of multiple linear regression models

We constructed linear models using a stepwise approach to explain the clinical score (i.e., MDS-UPDRS) for all subjects (i.e., for both the HC and PD groups). Multiple linear regression models were constructed using imaging features, genetic features, and two-way interactions between them. The multiple linear regression also considered family history information, MoCA, GDS, sex, age, and multi-center information as co-variants to control for confounding effects. Stepwise regression was applied to construct the regression models explaining the MDS-UPDRS scores^60^. An initial model with all possible independent variables along with interaction terms among them was constructed. Then, a candidate independent variable was removed if the removal of the variable led to a smaller p-value for the overall regression model. The process of variable removal was repeated until there was no improvement of the p-value of the regression model. For instance, the initial model considered DC values of four significant ROIs (from Table 1) and numerical SNP values of significant genetic variants (from Table 2) as independent variables. Then, each independent variable was tested for possible removal as described above. Subsequently, three linear regression models were constructed to explain the MDS-UPDRS score. The first model considered only neuroimaging features, the second model considered only genetic variants, and the third model considered both genetic variants and neuroimaging features:


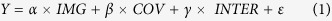



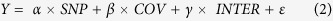



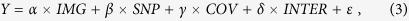


where Y is the clinical score; IMG is the imaging features; SNP is the genetic features; COV is the co-variants; INTER are the two-way interactions of predictors; α, β, γ and δ are estimated coefficients; and ε is a residual error term. The quality of multiple linear regression was assessed with adj-R^2^ values.

### Prediction and validation of the model

To validate the models, a leave-one-out cross validation method was applied by assigning each case as the test set and the remaining cases as the training set. The process was repeated 80 times, each time assigning a different test set. The performance of prediction was assessed with Pearson’s correlation coefficients between actual and predicted MDS-UPDRS scores. The RMSE was computed to quantify differences between the predicted and actual MDS-UPDRS.

## Additional Information

**How to cite this article:** Kim, M. *et al*. Imaging genetics approach to Parkinson’s disease and its correlation with clinical score. *Sci. Rep.* **7**, 46700; doi: 10.1038/srep46700 (2017).

**Publisher's note:** Springer Nature remains neutral with regard to jurisdictional claims in published maps and institutional affiliations.

## Supplementary Material

Supplementary Information

## Figures and Tables

**Table 1:** DC values of regions with significantly different structural connectivity in PD patients versus HC subjects.

Region	HC	PD	Corrected p-value
Associative cortex	0.017 ± 0.004	0.019 ± 0.004	0.047
Motor cortex	0.012 ± 0.003	0.015 ± 0.003	0.022
Thalamus	0.007 ± 0.002	0.008 ± 0.002	<0.001
Pallidum	0.006 ± 0.001	0.005 ± 0.001	0.007

DC values are reported as the mean ± standard deviation (SD). Corrected p-values are reported in the right-hand column.

**Table 2:** Genetic variants identified by imaging genetics analysis using neuroimaging features and clinical score as intermediate phenotypes.

Intermediate phenotype	Gene	SNP	Base-pair position	Minor allele	Empirical p-value
Associative cortex	PARK2	rs6901583	162543210	C	0.015
Motor cortex	PARK7	rs225103	8042301	T	0.043
Thalamus	SNCA	rs1473533	90735702	T	0.049
MDS-UPDRS	PARK2	rs9346876	162032871	C	0.034
	PARK2	rs9364608	162034799	G	0.034
	HtrA2	rs363611	74759708	T	0.044
	GIGYF2	rs7421653	233711471	G	0.048

**Table 3:** Detailed results of model A equation (1).

	Sum of squares	F-statistic	P-value	Pct. Exp. (%)
Associative cortex	682.07	8.65	0.004	5.78
Thalamus	1268.30	16.09	<0.001	10.75
Pallidum	980.81	12.44	<0.001	8.31
Family history	1023.50	12.98	<0.001	8.67
GDS	408.72	5.18	0.025	3.46
Age	190.98	2.42	0.124	1.62
Associative × Family history	710.76	9.01	0.004	6.02
Thalamus × GDS	609.60	7.73	0.007	5.16
Family history × Age	410.49	5.20	0.025	3.48
**Total**				53.25

Pct. Exp.: percentage of explained variance.

**Table 4:** Detailed results of model B equation (2).

	Sum of squares	F-statistic	P-value	Pct. Exp. (%)
rs6901583 (PARK2)	83.13	0.83	0.364	0.67
rs1473533 (SNCA)	136.13	1.36	0.247	1.10
rs9346876 (PARK2)	348.26	3.49	0.066	2.81
rs363611 (HtrA2)	703.45	7.04	0.009	5.68
GDS	303.08	3.03	0.085	2.45
Sex	523.84	5.24	0.025	4.23
rs1473533 × rs363611	1303.70	13.05	<0.001	10.52
rs9346876 × rs363611	1893.50	18.95	<0.001	15.28
**Total**				42.74

Pct. Exp.: percentage of explained variance.

**Table 5:** Detailed results of model C equation (3).

	Sum of squares	F-statistic	P-value	Pct. Exp. (%)
Motor cortex	472.94	10.30	0.002	4.64
Thalamus	699.97	15.24	<0.001	6.87
Pallidum	988.10	21.52	<0.001	9.70
rs6901583 (PARK2)	24.39	0.53	0.469	0.24
rs9346876 (PARK2)	649.43	14.14	<0.001	6.37
rs7421653 (GIGYF2)	135.18	2.94	0.091	1.33
rs363611 (HtrA2)	277.45	6.04	0.017	2.72
GDS	200.06	4.36	0.041	1.96
Sex	436.84	9.51	0.003	4.29
Age	228.45	4.98	0.029	2.24
Thalamus × rs7421653	430.90	9.38	0.003	4.23
Pallidum × rs6901583	246.29	5.36	0.024	2.42
Pallidum × Age	217.02	4.73	0.034	2.13
rs6901583 × rs363611	448.84	9.78	0.003	4.41
rs9346876 × rs7421653	132.27	2.88	0.095	1.30
rs9346876 × rs363611	927.71	20.20	<0.001	9.11
rs6901583 × GDS	496.61	10.82	0.002	4.87
rs363611 × GDS	375.28	8.17	0.006	3.68
**Total**				72.51

Pct. Exp.: percentage of explained variance.
