# Supplementary material for: Imaging genetics approach to Parkinson’s disease and its correlation with clinical score
Source: Sci Rep. 2017 Apr 21;7:46700. doi: 10.1038/srep46700 (PMC5399369; doi:10.1038/srep46700)
Supplement: Supplementary Information [file srep46700-s1.doc]

**SUPPLEMENTARY INFORMATION**

**Title: Imaging genetics approach to Parkinson’s disease and its correlation with clinical score**

Authors: Mansu Kim, Jonghoon Kim, Seung-Hak Lee and Hyunjin Park

**Additional analysis for 28 asymmetric onset PD patients**

We performed the same analysis, including linear model construction and prediction, for 14 right-sided and 14 left-sided onset cases in PD group using contralateral ROIs. We observed better results for the reduced 28 patient group compared to using the previous 40 patients in terms of adj-R^2^ values. *Model C*’s adj-R^2^ improved from 0.714 to 0.768, *model A*’s adj-R^2^ improved from 0.508 to 0.650, and *model B*’s adj-R^2^ improved from 0.422 to 0.485. Similar to the main results with 40 patients, *model C* (adj-R^2^ = 0.768) outperformed models A (adj-R^2^ = 0.650) and B (adj-R^2^ = 0.485) implying that combining genetic variants with neuroimaging features indeed was the best model. Further details are reported in supplementary Table S2, S3, and S4. Table S2 reports results of *model A* using only neuroimaging (similar to Table 3 in main Results). Table S3 reports results of *model B* using only genetic information (similar to Table 4 in main Results). Table S4 reports results of *model C* using both neuroimaging and genetic information (similar to Table 5 in main Results). Similar to the main results, for the reduced 28 patients, *model C* showed the best prediction performance with low RMSE (RMSE = 3.35) and a high correlation with the actual MDS-UPDRS (r = 0.847, p < 0.001) as shown in supplementary Figure S2. The prediction performance was better for the reduced 28 patients (r=0.847, RMSE=3.35) compared to main results of 40 patients (r=0.788, RMSE =7.82).

**Additional analysis for the PD patients**

We performed additional experiments to predict MDS-UPDRS scores just for the PD group. Consistent with the main results, the model combining genetic variants and neuroimaging features outperformed the models with genetic variants and neuroimaging features alone for the PD group. The model combining genetic and neuroimaging features predicted MDS-UPDRS with RMSE of 7.45 and showed a high correlation with the actual MDS-UPDRS (r = 0.763, p < 0.001) for the PD group. The model with only neuroimaging features showed a RMSE of 10.91 and a correlation of 0.466 (p = 0.002). The model with only genetic variants yielded a RMSE of 12.16 and a correlation of 0.462 (p = 0.003). Plots of the actual and predicted MDS-UPDRS scores for these three models are shown in the supplementary Figure S3.

**FIGURES**

**Supplementary Figure S1**. **Population stratification based on MDS analysis**. PPMI subjects were compared to the HapMap 3 reference population. The red dots represent PPMI subjects and the red circle represents the Caucasian population. Reference population in the HapMap 3 have been assembled: north Europeans (CEU), Africans (YRI), African in U.S.A. (ASW), Chinese (CHB), Chinese in U.S.A. (CHD), Japanese (JPT), Italian (TSI), America Indians (GIH), Kenya in Webuye (LWK) and Kenya in Kinyawa (MKK).


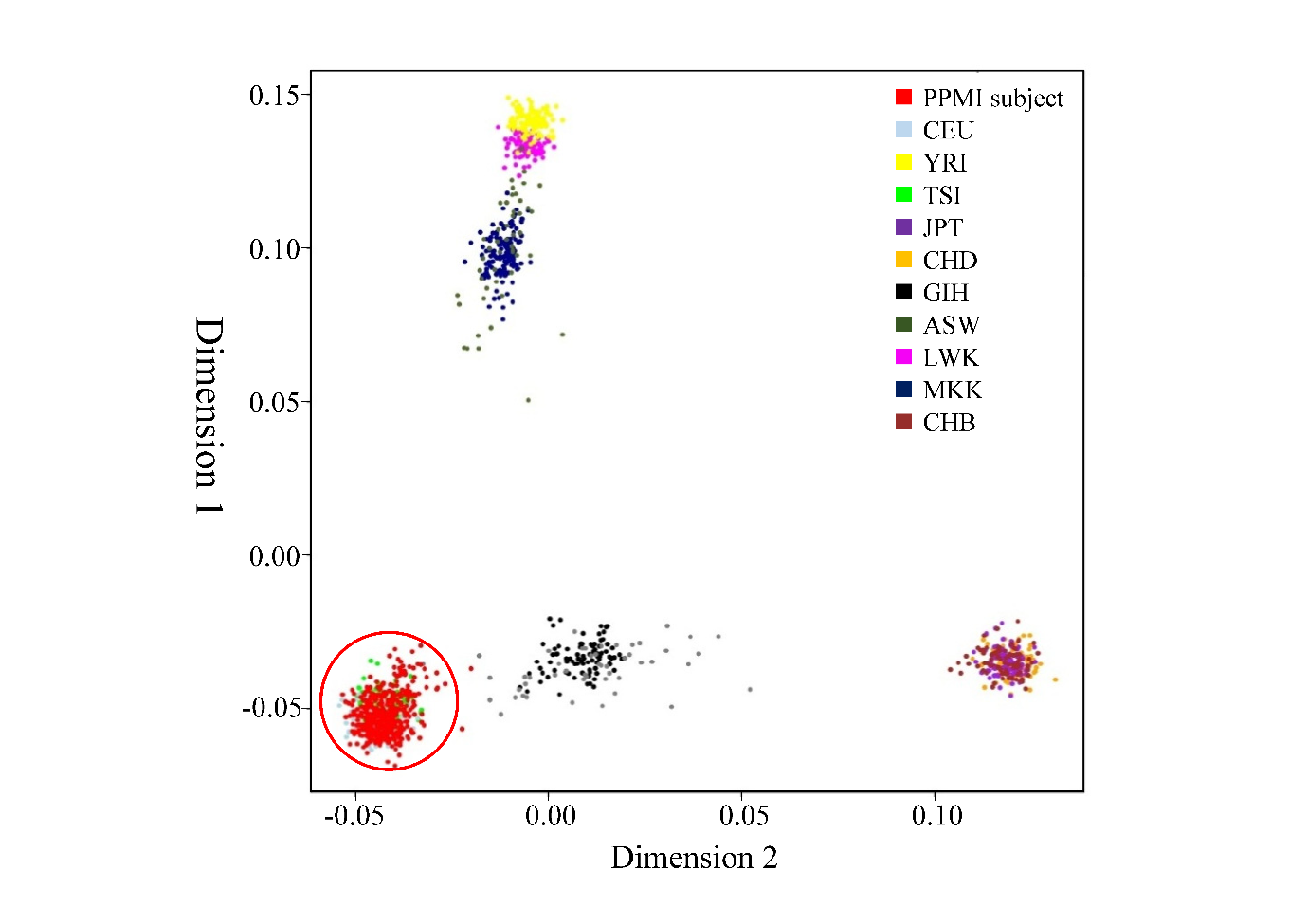


**Supplementary Figure S2.** Plots of actual and predicted MDS-UPDRS for three prediction models for 28 asymmetry onset patients. Sub-figure a), b), and c) show actual and predicted MDS-UPDRS of *Model A* (1)*, B* (2)*,* and *C* (3), respectively. The dashed line indicates the identity line (i.e., actual score = predicted score). Figure d) shows errors plots of the three models.


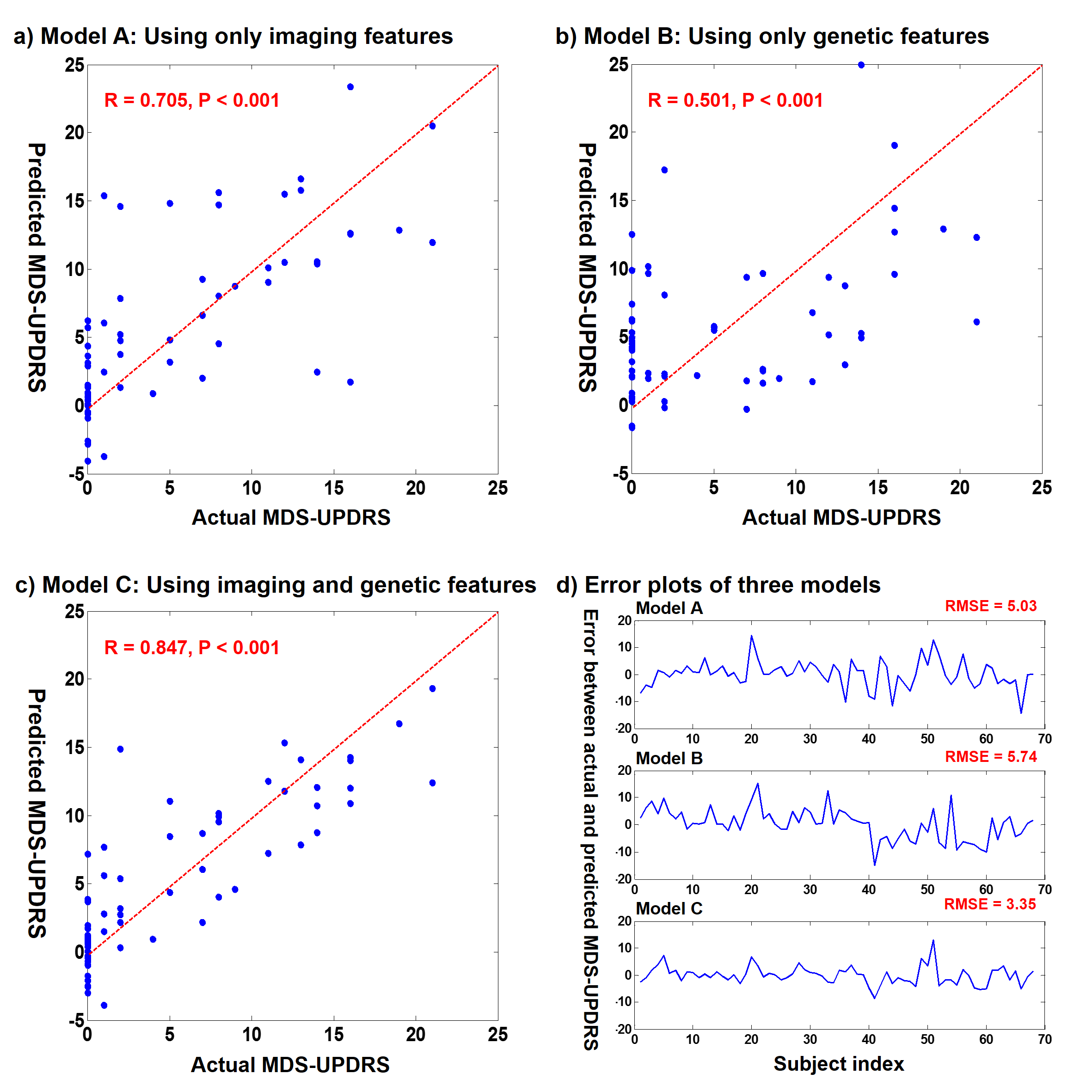


**Supplementary Figure S3.** Plots of actual and predicted MDS-UPDRS in three prediction models for only patients with PD. Sub-figure a), b), and c) show actual and predicted MDS-UPDRS of Model A (1), B (2), and C (3), respectively. Dashed line indicates identity line (i.e., actual score == predicted score). Figure d) shows errors plots of the three models.


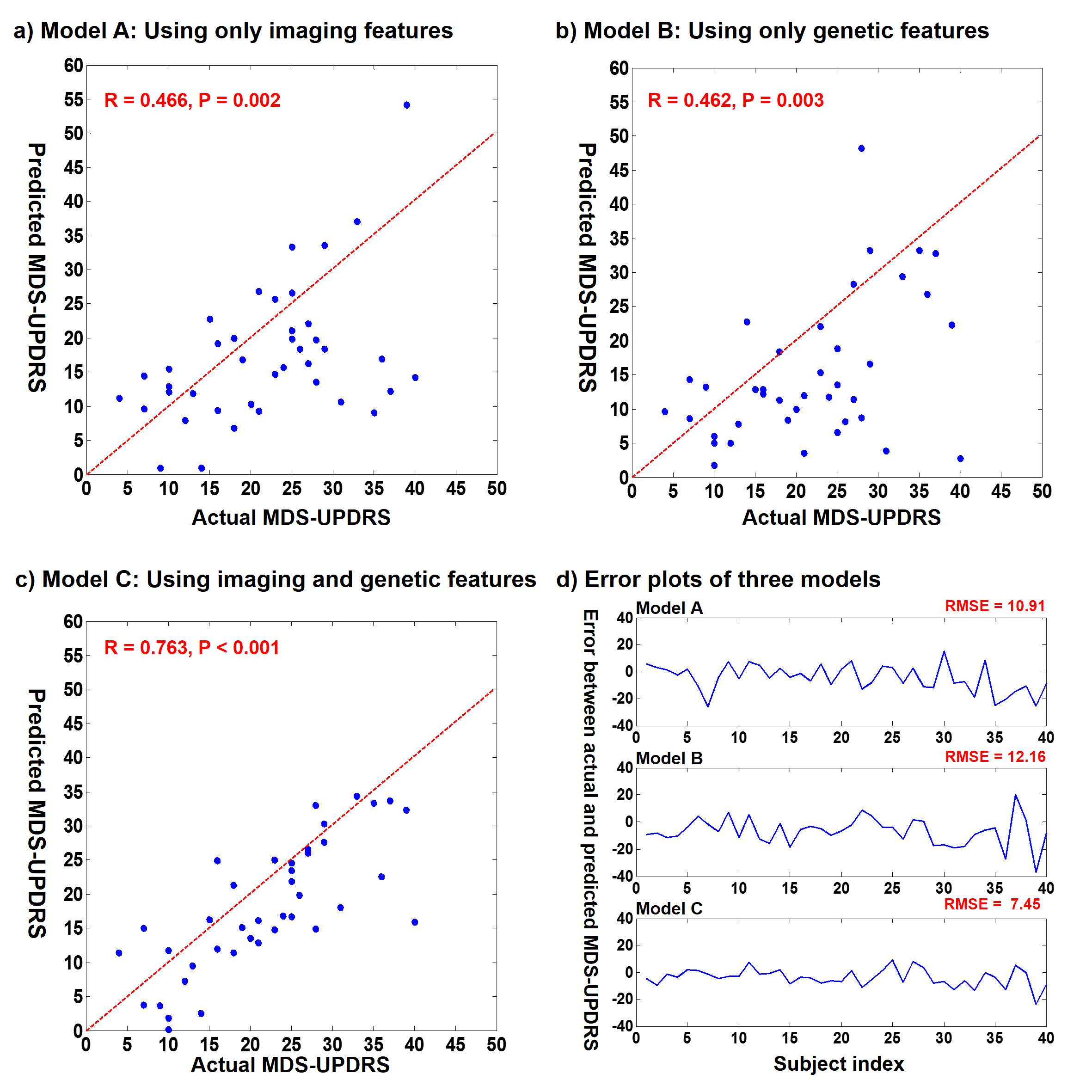


**TABLES**

**Supplementary Table S1. Participant information. Values are reported as mean ± standard deviation (SD) format. Among original 106 PD patients, 35 cases were left-sided, 41 cases were right sided, and 30 cases were bilateral onset cases. For the 40 chosen PD patients, 14 were right-sided, 14 were left-sided, and were 12 bilateral onset cases. The family history information was defined as the number of family members with PD. P-values were comparisons between HC and PD groups (not sub-groups within PD).**

|  | **HC** | **PD**  Total (right-sided, left-sided, bilateral onset) | **p-value** |
| --- | --- | --- | --- |
| Number of subjects | 40 | 40 (14, 14, 12) | - |
| Age (mean ± SD) | 62.2 ± 9.7 | 61.4 ± 8.8  (61.5 ± 9.3, 57.9 ± 7.9, 65.4 ± 8.2) | 0.704 |
| Sex (Male / Female) | 31 / 9 | 23 / 17  (9 / 5, 6 / 8, 8 / 4) | 0.057 |
| Disease duration  (mean ± SD) [month] | - | 7.92 ± 8.7  (10.1 ± 6.3, 2.4 ± 10.7, 11.2 ± 9.2) | - |
| MDS-UPDRS  (mean ± SD) | 0.4 ± 0.9 | 21.8 ± 9.4  (17.0 ± 6.9, 19.7 ± 9.5, 30.1 ± 6.4) | < 0.001 |
| Hohn and Yahn score  (mean ± SD) | - | 1.57 ± 0.5  (1.42 ± 0.51, 1.42 ± 0.51, 1.91 ± 0.28) | - |
| MoCA  (mean ± SD) | 28.0 ± 1.1 | 27.7 ± 1.9  (26.9 ± 2.5, 28.1 ± 1.6, 28.1 ± 1.1) | 0.314 |
| GDS  (mean ± SD) | 1.40 ± 2.5 | 2.05 ± 2.1  (1.4 ± 1.3, 1.6 ± 1.5, 3.2 ± 2.8) | 0.207 |
| Handedness  (Left/Right/ALL) | 4 / 32 / 4 | 2 / 37 / 1  (0 / 13 / 1, 1 / 13 / 0, 1 / 11 / 0) | 0.766 |
| Family history | 0.02 | 0.22  (0.21 ± 0.42, 0.28 ± 0.46, 0.16 ± 0.38) | - |

**Supplementary Table S2. Detailed results of model A for 28 asymmetric onset patients.**

|  | **Sum of squares** | **F-statistic** | **P-value** | **Pct. Exp.  (%)** |
| --- | --- | --- | --- | --- |
| Associative cortex | 310.93 | 8.32 | 0.006 | 5.13 |
| Thalamus | 759.94 | 20.34 | < 0.001 | 12.54 |
| Pallidum | 276.53 | 7.40 | 0.009 | 4.56 |
| Family history | 677.03 | 18.12 | < 0.001 | 11.17 |
| GDS | 72.74 | 1.95 | 0.169 | 1.20 |
| MOCA | 82.25 | 2.20 | 0.144 | 1.36 |
| Sex | 244.56 | 6.55 | 0.013 | 4.04 |
| Age | 7.35 | 0.20 | 0.659 | 0.12 |
| AssociativeⅹFamily history | 521.79 | 13.97 | < 0.001 | 8.61 |
| AssociativeⅹSex | 307.66 | 8.24 | 0.006 | 5.08 |
| Family historyⅹAge | 379.88 | 10.17 | 0.002 | 6.27 |
| GDSⅹSex | 227.63 | 6.09 | 0.017 | 3.76 |
| MOCAⅹAge | 175.20 | 4.69 | 0.035 | 2.89 |
| **Total** |  |  |  | 66.72 |

Pct. Exp.: percentage of explained variance.

**Supplementary Table S3. Detailed results of model B for 28 asymmetric onset patients.**

|  | **Sum of squares** | **F-statistic** | **P-value** | **Pct. Exp.  (%)** |
| --- | --- | --- | --- | --- |
| rs6901583 (PARK2) | 65.74 | 3.22 | 0.078 | 2.74 |
| rs1473533 (SNCA) | 26.80 | 1.31 | 0.257 | 1.12 |
| rs9346876 (PARK2) | 4.66 | 0.23 | 0.634 | 0.19 |
| rs363611 (HtrA2) | 53.91 | 2.64 | 0.110 | 2.25 |
| Fam | 92.02 | 4.51 | 0.038 | 3.83 |
| GDS | 43.56 | 2.13 | 0.150 | 1.81 |
| Sex | 231.64 | 11.35 | 0.001 | 9.65 |
| Age | 1.07 | 0.05 | 0.820 | 0.04 |
| rs6901583ⅹrs1473533 | 202.08 | 9.90 | 0.003 | 8.42 |
| rs6901583ⅹGDS | 175.84 | 8.62 | 0.005 | 7.33 |
| rs9346876ⅹrs363611 | 230.26 | 11.28 | 0.001 | 9.59 |
| GDSⅹSex | 84.98 | 4.16 | 0.046 | 3.54 |
| SiteⅹAge | 106.12 | 5.20 | 0.027 | 4.42 |
| **Total** |  |  |  | 54.94 |

Pct. Exp.: percentage of explained variance.

**Supplementary Table S4. Detailed results of model C for 28 asymmetric onset patients.**

|  | **Sum of squares** | **F-statistic** | **P-value** | **Pct. Exp.  (%)** |
| --- | --- | --- | --- | --- |
| Motor cortex | 51.80 | 5.62 | 0.022 | 3.25 |
| Thalamus | 0.12 | 0.01 | 0.910 | 0.01 |
| Pallidum | 221.04 | 23.98 | < 0.001 | 13.85 |
| rs6901583 (PARK2) | 8.16 | 0.89 | 0.351 | 0.51 |
| rs1473533 (SNCA) | 0.76 | 0.08 | 0.776 | 0.05 |
| rs9346876 (PARK2) | 35.04 | 3.80 | 0.057 | 2.20 |
| rs363611 (HtrA2) | 87.24 | 9.47 | 0.003 | 5.47 |
| GDS | 4.97 | 0.54 | 0.466 | 0.31 |
| Sex | 115.27 | 12.51 | 0.001 | 7.22 |
| Motor cortexⅹPallidum | 111.03 | 12.05 | 0.001 | 6.96 |
| ThalamusⅹPallidum | 51.99 | 5.64 | 0.021 | 3.26 |
| PallidumⅹSex | 86.68 | 9.40 | 0.003 | 5.43 |
| rs6901583ⅹGDS | 121.81 | 13.22 | 0.001 | 7.63 |
| rs1473533ⅹrs363611 | 87.91 | 9.54 | 0.003 | 5.51 |
| rs9346876ⅹrs363611 | 132.61 | 14.39 | < 0.001 | 8.31 |
| **Total** |  |  |  | 69.96 |

Pct. Exp.: percentage of explained variance.
